# Text Analysis and Policy Guidance of Emotional Intonation of Enterprise Management Based on Deep Learning

**DOI:** 10.1155/2022/3428078

**Published:** 2022-08-30

**Authors:** Ni Yang, Jing Qiu

**Affiliations:** School of Accounting, Guizhou University of Finance and Economics, Guiyang 550025, China

## Abstract

In the context of global science and technology, all countries pay more and more attention to the text analysis of emotional intonation, and the emotional intonation text analysis and policy orientation of enterprise management in major international and domestic enterprises have also changed from shallow to deep. In the twenty-first century, with the rapid development of human society, people's demand for living standards and material needs increases rapidly, and employees' awareness and needs for work are constantly changing. At present, there is the problem of emotional intonation text analysis error in the management of the enterprise, and the task and emotional transmission command are not clear and thorough. It is necessary to reasonably use deep-learning-related algorithms, especially convolutional neural network and other algorithms, to study the emotional intonation text analysis and policy guidance of the enterprise management. Aiming at the forefront of deep learning development, the latest deep learning technologies are constantly introduced. The research field of emotional intonation text analysis and policy orientation of enterprise management is focused. Through simulation experiment, the characteristics of emotional intonation text analysis and policy orientation research of different enterprise management are compared and analyzed, so as to further improve the emotional intonation text analysis and policy orientation of deep learning for enterprise management.

## 1. Introduction

Can Chinese capital market investors understand the management's “commitment”? This paper uses LSM deep learning technology to analyze the management response in the annual performance briefing of Chinese listed companies and discusses the actual semantics of management. The results show that investors can understand the true semantics of management, and the capital market gives a positive response to the positive semantics and gives a more timely and meaningful negative response to the negative semantics. Starting from a three-factor model, we further investigate whether management semantics is a factor in the investor stock trading strategy, but there is no evidence that investors use management semantics as a factor in the trading strategy. On the one hand, this study shows that nonfinancial disclosure channels such as performance briefing are valuable, verifying the perspective theory of behavioral economics; on the other hand, it is worth extending to other areas of text analysis for future application of [1]. With the development of deep learning, more and more neural network-based algorithms are used for classifying text sensation, and the accuracy of classification is constantly improved. If we blindly pursue accuracy and go deep into the network level, it will bring huge obstacles to the reaction performance in real application scenarios. By studying techniques such as integrated representation of texts, we can further grasp the main features of the text in the classification logic based on the current FastText models. A novel weight-based WDFT model is presented for classifying lighter text immunity, achieving high accuracy while ensuring the simplicity of the model and better solving the classification task of text immunity. To solve the problem of employee behavior analysis in key positions, a video-based behavior analysis method is proposed. Multipostural sampling employee behavior records were created, formed by the YOLOv3 network, yielding a behavior detection model [2]. The proposed behavior analysis algorithm is combined with the behavior detection model. Based on the behavior analysis algorithm and the similarity and brightness characteristics of the image, the evaluation results of mobile phone shutdown, sleep, and game events are presented. The experimental results show that the product data group has high recognition accuracy in identifying employee behavior and behavior analysis, which can handle in real time [3].

Emotional text analysis mainly uses text extraction technology to analyze emotional processing, and identifying the tendency of subjective text is a positive, negative, and neutral process. This classification of text emotion particles is insufficient, incomplete, too rigid, and violent, which not only effectively reflects the intensity and size of different emotion particles in the text but also requires extensive manual annotation. We verify the effectiveness of emotion word carrier and improve the accuracy of text emotion classification [4]. Through a sample survey of nonfinancial enterprises that issued bonds in China's stock market in 2017, this paper studies the influence of accounting text intonation on bond communication, establishes an emotional dictionary for China's financial market, and quantifies the direction intonation by using text extraction technology. The study shows that management's positive tone can significantly reduce the credit spread of bonds. The management tone has a less negative relationship with the bond interest rate difference. In addition, the bond market is more sensitive to the tone of state-owned and small-business management [5]. Selecting listed companies from 2008 to 2019, through management analysis and discussion text analysis, this paper studies the constraints and impact mechanism of management emotional voice signals on corporate financing; the results show that management of positive net tone may have signal conduction effect, alleviate enterprise financing constraints, by expanding equity financing channels to achieve this effect, attract the attention of investors, is the management emotional influence to financing bottleneck mechanism. The results of the heterogeneous effects of speech tampering strategies show that investors cannot effectively identify text readability strategies and positive speech bias strategies, while speech splitting strategies are fully understood by investors, leading to differences in the emotional voices of people at different levels of management in reducing financial constraints. Expand the voice. This paper confirms that management can effectively use the emotional sound in the text to exert the signal transmission effect and guide the market to achieve its ideal goal [6].

If the hardware is the body of the company, the software is the blood of the company; the invisible emotion and emotion cannot be touched but really exist in the company that is the “gas” of the company. On the contrary, mismanaged corporate emotions and emotions, creating a healthy entrepreneurial spirit, and psychological and emotional air are major issues that managers cannot face up to and think about. Traditional management theory holds that human feelings have an influence on the realization of organizational goals in [7]. The development and evolution of network public opinion between the COM incident on June 6 and the Hyderabad incident were compared, and the ways of emotional development of netizens were revealed through the collection and analysis of microblog data, especially the emotional analysis of microblog text. Comparing the gains and losses of the two online incidents to deal with these events, we summarize the strategy of enterprises to effectively deal with the network public opinion, which will help enterprises to deal with the public opinion crisis online [8].

In the 1980s, emotion management as a management model was proposed by many experts and scholars. In the twenty-first century, with the rapid development of human society, people's demand for living standards and material needs increases rapidly, and employees' awareness and needs for work are constantly changing. This paper briefly introduces the concept and significance of emotion management, summarizes the main factors affecting employees “emotions, analyzes the current situation of employees” emotions in the chemical industry, and puts forward some suggestions for emotion management [9]. Based on the on-site interviews and two questionnaires of 28 middle and senior managers, as well as the analysis of exploratory factors and confirmed factors, this paper formulated the scale to measure the emotional connection of managers in the transformation process of local enterprises in China. The results show that managers' emotional attachment consists of three factors: attachment to life, attachment to work and situation, and attachment to life that are positively related to attachment to work. They also have a positive relationship with context-binding. The research content not only illuminates the emotional culture of the organization but also provides a new way to promote the transformation of enterprises through emotional strategies [10].

Structural and functional analysis of managers' trust found that managers need to handle three trust relationships in their careers: subordinate trust, supervisor trust, and colleague trust. These three trusts lead to superior support, subordination, and peer cooperation, which is conducive to improving the personal performance of business operators. In modern enterprise management, the emotional contribution of employees to enterprise development is very important. This paper discusses the concept of emotion management and its role and position in human resource management [11]. By utilizing the “emotional talent” of employees, we can grasp the emotional management method in management practice, promote the emotional investment of employees, and ensure the continuous success of [12]. With the increasing popularity of online review and suggestion systems, emotion analysis has gradually become an important academic research task that has attracted the attention of many scholars in recent years. The traditional method to solve the problem of text emotion analysis is mainly based on the emotional dictionary or shallow knowledge, and the feature extraction and classification are conducted through regression and classification schemes. However, due to the short and clear features of the annotated text, a large number of unknown words make these methods have data scarce and ignore the problem of word tracking. Starting from these methods, deep learning methods are used to analyze the mood of comment text, and we can find a deep understanding of human emotional expression mode through comparative combination experience [13].

In recent years, public opinion sentiment analysis has become one of the most popular topics in the NLP space. The sentiment analysis and emotional trend assessment of these public opinion data provide important support for the company government to make strategic decisions. At present, the supervised plane automatic learning algorithm model is widely used in emotion analysis research. The manual annotation process is lengthy, and the annotation results often do not reflect the real situation of the data. This paper expounds on the current situation of emotional word analysis, discusses the emotional analysis of public opinion data by using deep learning and fusion methods, summarizes the shortcomings and challenges of Chinese emotional analysis, and looks forward to the future [14]. Some scientists say that deep learning algorithms are a gem in the crown of artificial intelligence. By applying deep neural network (DNN) models, deep learning has become the closest intelligent learning method to the human brain. Not only Google, Baidu, and other companies participate in the application research of in-depth learning but also e-commerce giants such as Jingdong and Ali join the competition. This paper introduces the in-depth study of e-commerce in China [15].

## 2. Deep-Learning-Related Algorithms

### 2.1. Artificial Neural Network

Artificial neural networks are the foundation of deep learning, simulating human brain models. Neuron is the basic computational unit of deep learning model [16]. The neuron output formula is

(1) y=f∑i=1nwix−iθ,

where *f* represents the activation function. Commonly used activation functions are the Sigmoid activation function, the Tanh activation function, and the ReLU activation function. The respective representation formulas are as follows:

(2) $$\begin{array}{c} f\left(z\right)=\frac{1}{1+\exp\left(-z\right)},\\ f\left(z\right)=\frac{e^{z}-e^{-z}}{e^{z}+e^{-z}},\\ f\left(z\right)=\max\left(0,z\right). \end{array}$$

### 2.2. Circular Neural Network and Long-and Short-Term Memory Network

The recurrent neural network can handle the time-series data well. The simplest recurrent neural network formula is as follows:

(3) $$\begin{array}{c} o_{t}=g\left(V•S_{t}\right),\\ S_{t}=f\left(U•X_{t}+W•S_{t-1}\right). \end{array}$$

However, as the input increases, the standard RNN decreases the perception ability of the data nodes, resulting in the gradient disappearance and gradient dispersion phenomenon. To address this problem, the transformation function is defined as follows:

(4) $$\begin{array}{c} i_{\left(t\right)}=\sigma \left(W_{xi}^{T}\cdot x_{\left(t\right)}+W_{hi}^{T}\cdot h_{\left(i-1\right)}+b_{i}\right),\\ g_{\left(t\right)}=\tanh\left(W_{xg}^{T}\cdot x_{\left(t\right)}+W_{hg}^{T}\cdot h_{\left(t-1\right)}+b_{g}\right),\\ f_{\left(t\right)}=\sigma \left(W_{xg}^{T}\cdot x_{\left(t\right)}+W_{hf}^{T}\cdot h_{\left(t-1\right)}+b_{f}\right),\\ c_{\left(t\right)}=i_{\left(t\right)}⊗g_{\left(t\right)}+f_{\left(t\right)}⊗c_{\left(t-1\right)},\\ o_{\left(t\right)}=\sigma \left(W_{xo}^{T}\cdot x_{\left(t\right)}+W_{ho}^{T}\cdot h_{\left(t-1\right)}+b_{o}\right),\\ h_{\left(t\right)}=o_{\left(t\right)}⊗\;\tanh\left(c_{\left(t\right)}\right), \end{array}$$

*σ*where *σ* is the sigmoid activation function.

### 2.3. Support Vector Machine

The core idea of the SVM algorithm that is fast and reliable is to find an optimal classification hyperplane [17] in high-dimensional space. In linearly separable samples, the formula for defining the hyperplane is

(5) $$\begin{array}{c} w^{T}x+b=0. \end{array}$$

The distance from any point in the sample to the optimal plane is

(6) $$\begin{array}{c} r=y_{0}\left(\frac{w}{\left\|w\right\|}x_{0}+\frac{b}{\left\|w\right\|}\right). \end{array}$$

Any training data satisfies the following formula:

(7) $$\begin{array}{c} y_{0}\left(w^{T}x_{0}+b\right)\geq 1. \end{array}$$

So the SVM solves the constrained optimization problem as follows:

(8) $$\begin{array}{c} \underset{w,b}{\min}\frac{1}{2}{\left\|w\right\|}^{2},\\ s.t.y_{i}\left(w^{T}x_{i}+b-1\right)\geq 0\;i=1,2,\ldots .,N. \end{array}$$

Cross-validation methods and prior knowledge can be used to select the kernel functions that meet the data distribution. The following are the four common kernel functions:

(1) Linear kernel function
  (9) $$\begin{array}{c} Κ\left(x,x_{i}\right)=x\cdot x_{i}. \end{array}$$
(2) Polynomial kernel function
  (10) $$\begin{array}{c} Κ\left(x,x_{i}\right)={\left(\left(x\cdot x_{i}\right)+b\right)}^{d}. \end{array}$$
(3) Gaussian kernel function
  (11) $$\begin{array}{c} Κ\left(x,x_{i}\right)=\exp\left(-\gamma {\left\|x-x_{i}\right\|}^{2}\right). \end{array}$$
(4) Sigmoid kernel function
  (12) $$\begin{array}{c} Κ\left(x,x_{i}\right)=\tanh\left(\gamma x\cdot x_{i}+b\right). \end{array}$$

### 2.4. Logical Regression

The logic regression algorithm is very simple and practical, belongs to the generalized linear regression model, and is often used to solve the binary classification problem [18]. The basic mathematical formula is as follows:

(13) $$\begin{array}{c} g\left(x\right)=\frac{1}{1+e^{-x}}. \end{array}$$

Mathematical expression model of logistic regression is as follows:

(14) $$\begin{array}{c} h_{\theta }\left(x\right)=g\left(\theta^{T}x\right)=\frac{1}{1+e^{-\theta^{T}x}}. \end{array}$$

For binary classification problems, the logistic regression formula can be simplified as follows:

(15) $$\begin{array}{c} p\left(y=1|x;\theta \right)=\frac{1}{1+e^{-\theta^{T}x}},\\ p\left(y=1|x;\theta \right)=1-\frac{1}{1+e^{-\theta^{T}x}}. \end{array}$$

For a single sample, the model assumes a unity of

(16) $$\begin{array}{c} P\left(y|x;\theta \right)={\left(\frac{1}{1+e^{-\theta^{T}x}}\right)}^{y}{\left(1-\frac{1}{1+e^{-\theta^{T}x}}\right)}^{1-y}, \end{array}$$

where *y* ∈ {0,1}.

## 3. Text Analysis of Emotional Intonation

Text emotion analysis is one of the studies of natural language processing. Pang et al., in 2002, has been widely studied and applied in data exploration, information search, text search, and other fields. As textual emotion analysis plays an important role in society and the economy, it transforms into an interdisciplinary research topic. In recent years, high-level academic conferences at home and abroad have produced many excellent research results. In China, Internet companies such as Baidu and Ali have provided users with a text sentiment analysis API to help users quickly deploy online applications and reduce development costs. Lip, in 2012, introduced the emotional analysis in detail. According to the research method, the text emotion analysis method can be divided into supervised and nonsupervised methods. In foreign countries, the research of English text emotional analysis is gradually mature. The main methods of text sentiment analysis can be divided into sentiment dictionary based sentiment analysis method, sentiment analysis method based on traditional machine learning and sentiment analysis method based on deep learning. The method uses processed and labeled data to form a classifier and then uses a classifier to assess the emotional trend [19] of unlabeled data.

The emotional dictionary mainly consists of emotional polarity or emotional intensity. There are three common methods to create emotional dictionaries: methods based on manual annotation, methods based on existing dictionaries, and methods [20] based on body statistics. The research on the construction of emotion dictionaries abroad started earlier, among which SentiWordNet is the most seriously affected English emotion dictionary. In China, the research on the Chinese emotion dictionary started relatively late. Founded by Dong Zhendong and Dong Qiang, HowNet was a widely used emotional dictionary in early China. Manual creating emotional dictionaries requires a lot of manpower and material resources, and their effects vary by field, so researchers tend to create emotional dictionaries automatically. Hu cites emotionally inclined words as opening phrases and generates a general emotional dictionary through constant iterations. Emotional polarity reverses only when words change. Huang et al. first mark the emotional polarity of high-frequency words in the main text of a specific domain and then use connectors to evaluate the emotional polarity between words to generate a domain-specific emotion dictionary. Due to the few Chinese knowledge bases, most domestic scholars will combine different methods of [21] when automatically compiling the Chinese sentiment dictionary. Zhou Yongmei adopted the Chinese-English translation method, searched for the English words translated by the HowNet dictionary in the SentiWordNet dictionary, calculated the average emotional intensity of all the words, and finally generated the Chinese emotional dictionary. Wang Changhou et al. used the model-based paddle method to extract emotional words, through which they collected a large number of words not included in the traditional emotional dictionary.

This is a popular research method that applies traditional machine learning methods to text emotion analysis. It can learn training data sets to extract functions and generate a text emotion analysis model [22]. Experience has shown that analyzing text sensation using SVMs is effective. Zhang adopted the Bayesian algorithm weighted SME TF-DF own value, applied it to the emotional analysis of the course evaluation text, and achieved good results. Chiong et al. analyzed the text sense of new finance using an SVM-based approach and used a particle test optimization algorithm to optimize the parameters. Huang et al. used the Stanford method to support the voice-dependent support vector machine to analyze financial information, with good results. Liu Siye et al. introduced the keywords of facial expression to expand the text characteristics of tourists' microblog and adopted the maximum entropy model to achieve good results.

In the early 1990s, the development of scientific and industrial deep learning was directly suspended due to the problem of gradient disappearance in the BP algorithm. Until 2006, Hinton, the father of deep learning, has always proposed the weight initialization scheme through the fine adjustment of unsupervised training and supervised training, thus solving the problem of gradient disappearance in the process of deep network formation. The emotional dictionary method and traditional machine learning method have produced many excellent research results in the research of emotional text analysis, but a large labor cost of [23] is needed early in the data processing process.

Deep learning is a machine learning technique based on a set of self-learning algorithms and deep neural networks. In recent years, with the rapid development of technology, the research and application of in-depth learning have also undergone great changes. Compared with traditional machine learning methods, deep learning reduces artificial factors, has deeper model depth, mimics the mechanism by which the human brain processes data, and is able to deeply extract data features. A convolutional neural network is a special neural network that has achieved very good [24] in the field of computer vision. The convolutional layer of the convolutional neural network can extract the local features very well. Six sets of data were used for experimental comparison, demonstrating that TextCNN is effective for some simple text classification tasks. Yu et al. have studied the problem of cross-domain sentence fitting analysis, designing a model involving two distinct networks of convolutional neurons that learn two hidden feature representations from labeled and unlabeled data. The emotional classification, using low-monitoring convolutional neural networks, was performed by Guan et al. The framework consists of two stages: the first is to learn the representation of a sentence, which is not adequately monitored, and the second is to perfect the sentences with labels. Zhao et al. proposed a text sentiment classification method by using the underlying contextual semantic relations and statistical features of co-occurring words on Twitter, combined with a powerful neural network.

Long-term memory networks contribute to natural language modeling, which is important for computer processing and computer processing natural language, deep into the semantic comprehension level [25]. Twitter emotions were classified using long, short-term memory networks. Huang et al. suggest encoding syntactic knowledge into long- and short-term tree memory networks to improve the expression of sentences and phrases. Learning emotion intensity using long- and short-term bidirectional memory networks. Long- and short-term memory networks of language regulation for textual emotion analysis were proposed by Qian et al. This model combines emotional dictionaries, negative words, and intensity words and more accurately captures feelings in sentences. Fu et al. introduced a dictionary-modified LSM model that uses the emotion dictionary to train a word sense classifier to maintain the emotional integration of each word and address the problem that word integration contains more semantic information than emotional information. Lu et al. used the word integration model CBOW to capture word semantic features in microblog text sentiment analysis and extract deep sequence word vector features using stacking BiLSTM.

## 4. Example Analysis

### 4.1. Experimental Preparation

Based on the experiment designed for the emotion intonation analysis of enterprise management based on deep learning, we should first collect and classify the emotion tendency data and then analyze it. The experimental environment and configuration adopted in this article are shown in the following Table 1.

**Figure 1:**
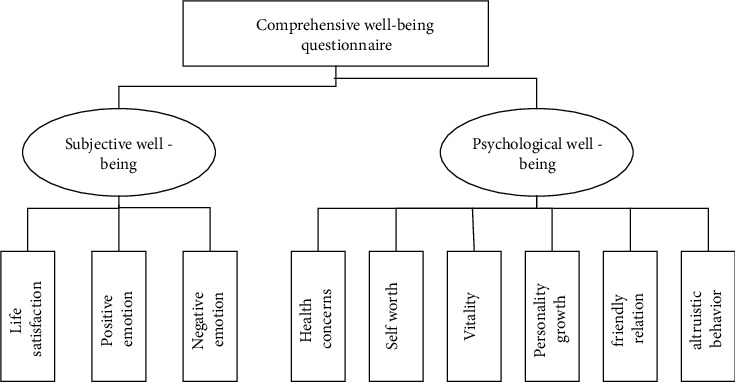
The comprehensive happiness questionnaire.

Based on the management staff, a comprehensive happiness questionnaire was specially designed as the measurement questionnaire of this study, and the emotional text analysis of the enterprise management personnel was used in two modules and nine dimensions. It is shown in Figure 1 and Table 2.

Descriptive statistics, factor analysis, correlation analysis, and regression analysis were used in this study. With the enterprise managers, the enterprise managers of all levels in Chengdu, Shenzhen, Xiamen, and Wuhan were investigated. The enterprises mainly include finance, logistics, construction, and other aspects. The sample was selected in April 2022. A total of 300 questionnaires were distributed through the Internet, and 237 valid questionnaires were collected. The sample is provided in Table 2.

### 4.2. Statistical Results of the Scale

**Figure 2:**
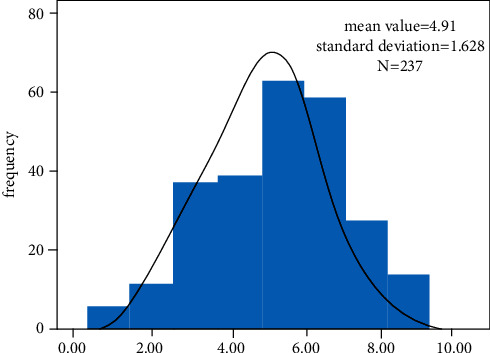
Distribution of the happiness index.

In the general happiness questionnaire, the happiness index scores ranged between 4.91 and 1.628, with the highest proportion being “mean” (see Figure 2). The lowest scores were negative emotions (2.42, 0.96), and the highest scores were self-worth (5.25, 68.4). Besides negative emotions, other dimensions were scored between 3.91 and 5.68 (see Figure 2).

**Figure 3:**
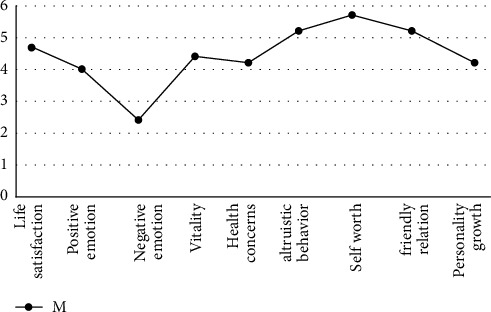
Mean average of each dimension of happiness.

**Figure 4:**
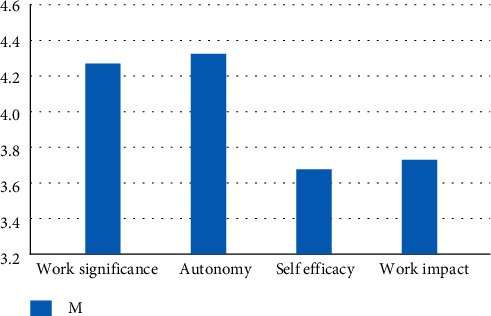
Bar chart of the average value of all dimensions of psychological authorization.

**Figure 5:**
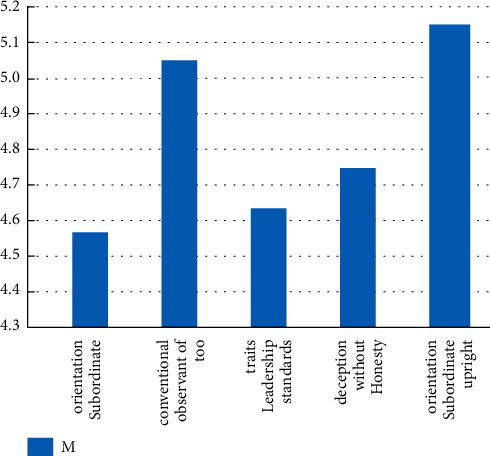
Bar chart of the mean of each dimension.

Figure 3 shows that the values for all well-being dimensions are type V, and the lowest point is negative effect. Understandably, Figure 4 respondents' happiness, Figure 5, in all aspects are positive and high.

### 4.3. Analysis and Comparison of Experimental Results

#### 4.3.1. Mean Comparison of Each Dimension

1. According to the survey results of the psychological authorization scale, the average of the four dimensions of psychological authorization is shown in Figure 4, where the autonomy dimension has the highest score of 4.42 + 0.79; self-efficacy and work impact are the lowest, 3.71 ± 0.76 and 3.71 ± 0.90, respectively. It can be seen that the mean for each dimension is between “uncertain” and “very consistent.”
2. According to the survey results of the integrity management scale, the average score of the five dimensions of integrity management is shown in Figure 5. We can see that integrity and selflessness have the highest score of 5.17 + 0.91, while subordinates have the lowest score of 4.59 + 0.93. The various dimensions of integrity management were scored between 4.59 ± 0.93 and 5.17 ± 0.91, namely between “slightly consistent” and “fully consistent.”

### 4.4. Experimental Summary

#### 4.4.1. Innovation of This Experiment

1. As a questionnaire compiled by domestic scholars, the comprehensive happiness questionnaire has high reliability and validity and has been verified in different groups. However, the questionnaire was not applied to business managers prior to this study. In this study, the volume was used for the first time for business managers to conduct qualitative and quantitative analysis, resulting in good credit validity and expanding the scope of the comprehensive well-being questionnaire.
2. According to the results of this study, the purpose is to adjust the psychological empowerment of management, improve the well-being of enterprise managers, and stimulate their potential through the intermediary variables of well-being. In order to improve the manager's ability of honesty management and manage the enterprise and staff better, the theory of honesty management and the theory of humanistic management are expanded.

#### 4.4.2. Limitations of This Experiment

1. Due to time, resources, and financial constraints, the sampling range of this paper is very limited, and the representativeness of the sample data needs to be further tested by more researchers. Because the mechanisms of happiness are very complex and the influencing factors are also very complex, they are greatly influenced by regional and economic conditions. Therefore, it is suggested that future studies can expand the regional range of the sample and increase the volume.
2. This study focuses on model building and the surface analysis of demographic variables. Furthermore, this study only collected and analyzed data on demographic variables from four aspects: gender, education, years of work, and position. Other demographic variables may differ significantly. No more research was done in this paper due to energy and condition limitations, which require further discussion by later researchers.

## 5. Conclusion

In view of the emotional intonation text analysis and policy orientation of enterprise management, the technical methods of the deep learning are preliminarily introduced, but more in-depth research is still needed. The application of deep learning method, especially the network neural method; has just started in the field of emotional intonation text analysis and policy orientation of enterprise management; and faces many uncertainties and challenges, but the application prospect is broad.

1. Aiming at the forefront of deep learning development, the latest deep learning technologies are constantly introduced. The research field of emotional intonation text analysis and policy orientation of enterprise management is focused. Through simulation experiment, the characteristics of emotional intonation text analysis and policy orientation research of different enterprise management are compared and analyzed, so as to further improve the emotional intonation text analysis and policy orientation of deep learning for enterprise management.
2. In the face of doubts about emotional intonation text analysis and policy-oriented research, conduct the interpretability research of deep learning. Based on domain knowledge, an interpretable deep learning model is established to promote the development of crossover research between emotional intonation text analysis and policy-oriented research in the field and the field of deep learning research.
3. Enterprise management can not only improve the enthusiasm of enterprise management but also bring positive impact on the health of employees by using emotional tone text analysis and policy-oriented research and analysis to select the optimal scheme.

## Figures and Tables

**Table 1:** Experimental environment and configuration.

Experimental environment	Configuration information
Operating system	Windows10 (64 bit)
Processor	Intel® Core™ i7-4790, 3.6 GHz
Internal storage	16 GB
Programming language	Python 3.6
Deep learning framework	TensorFlow 1.13
Partition tool	Jieba, PkuSeg

**Table 2:** Description of the sample situation.

Demographic variables	Encoding	Class	Number of people	Percentage (%)	Cumulative percentage (%)
Sex	0	Man	158	66.7	66.7
	1	Woman	79	33.3	100

Education level	1	Specialist below	18	7.6	7.6
	2	Junior college education	53	22.4	30
	3	Undergraduate course	149	62.9	92.8
	4	Master's degree or above	17	7.2	100

Working life	1	1–3 Years	26	11	11
	2	3–5 Years	107	45.1	56.1
	3	5–10 Years	69	29.1	85.25
	4	More than 10 years	35	14.8	100

Position	1	Grassroots management personnel	62	26.1	26.1
	2	Middle management	142	59.9	86.1
	3	Senior management staff	33	13.9	100

## Data Availability

The experimental data used to support the findings of this study are available from the corresponding author upon request.
